# Safety and Efficacy of Dapagliflozin in Patients with Heart Failure with Reduced Ejection Fraction: Multicentre Retrospective Study on Echocardiographic Parameters and Biomarkers of Heart Congestion

**DOI:** 10.3390/jcm13123522

**Published:** 2024-06-16

**Authors:** Ilaria Battistoni, Giulia Pongetti, Elena Falchetti, Irene Giannini, Roberto Olivieri, Filippo Gioacchini, Paolo Bonelli, Daniele Contadini, Lorena Scappini, Marco Flori, Andrea Giovagnoli, Renata De Maria, Marco Marini

**Affiliations:** 1 Department of Cardiovascular Sciences, Clinic of Cardiology, Ospedali Riuniti, 60100 Ancona, Italy; giuliapongetti@gmail.com (G.P.); marcomarini1975@gmail.com (M.M.); 2 Cardiology Department, Senigallia Hospital, 60019 Senigallia, Italy; elena.falchetti@yahoo.it; 3 Department of Cardiology, Camerino-Hospital, 62032 Camerino, Italy; ire_gia@hotmail.com; 4 Cardiology Unit, Ospedali Riuniti Marche Nord, 61121 Pesaro, Italylorena.scappini@gmail.com (L.S.); 5 O.U. of Cardiology, Ospedale Civile A. Murri, 63900 Fermo, Italy; 6 Department of Cardiology, Cardiac Intensive Care Unit, Centre of Telemedicine, Italian National Research Centre on Aging (INRCA), 60127 Ancona, Italy; paolo7bonelli@gmail.com; 7 Cardiology Division, Ospedale Provinciale AREA VASTA 3, 62100 Piediripa, Italy; daniele.contadini@gmail.com; 8 U.O.C. Cardiology-Utic Ospedale della Misericordia Urbino AST Pesaro-Urbino, 61029 Urbino, Italy; mflori3001@gmail.com; 9 Cardiology Unit, Carlo Urbani Hospital, 60035 Jesi, Italy; giovyand@libero.it; 10 Istituto di Fisiologia Clinica CNR Pisa, 56124 Pisa, Italy; demaria05790@gmail.com

**Keywords:** heart failure, dapaglifozin, SGL2i, real-world

## Abstract

**Objectives**: Dapagliflozin has shown efficacy in clinical trials in patients with heart failure and reduced ejection fraction (HFrEF). However, real-world data on its use and outcomes in routine clinical practice are limited. We aimed to evaluate the utilisation and safety profile of dapagliflozin in a real-world population of HFrEF patients within the Marche region. **Methods**: Nine cardiology departments within the Marche region retrospectively included HFrEF patients who were initiated on dapagliflozin therapy in an outpatient setting. Data on medical history, comorbidities, echocardiographic parameters, and laboratory tests were collected at baseline and after 6 months. Telephone follow-up interviews were conducted at 1 and 3 months to assess adverse events. We defined the composite endpoint score as meeting at least 50% of four objective measures of improvement among: weight loss, NYHA decrease, ≥50% Natriuretic peptides (NP) decrease, and guideline/directed medical therapy (GDMT) up titration. **Results**: We included 95 HFrEF patients aged 66 ± 12 years, 82% were men, 48% had ischemic heart disease, and 20% had diabetes. At six months, glomerular filtration rate declined (*p* = 0.03) and natriuretic peptides levels decreased, on average, by 23% (*p* < 0.001). Echocardiographic measurements revealed a decrease in pulmonary artery pressure (*p* < 0.001) and E/e’ (*p* < 0.001). In terms of drug therapy, furosemide dosage decreased (*p* = 0.001), and the percentage of the target dose achieved for angiotensin receptor–neprilysin inhibitors increased (*p* = 0.003). By multivariable Cox regression, after adjustment for age, sex, the presence of diabetes/prediabetes, and HF duration, higher baseline Hb concentrations (HR 1.347, 95% CI 1.038–1.746, *p* = 0.025), and eGFR levels (HR 1.016, 95% CI 1.000–1.033, *p* = 0.46). **Conclusions**: In a real-life HFrEF population, dapagliflozin therapy is safe and well-tolerated, improves echocardiographic parameters and biomarkers of congestion, and can also facilitate the titration of drugs with a prognostic impact.

## 1. Introduction

Heart failure (HF) represents a significant burden on global healthcare systems, with high morbidity and mortality rates [1]. Despite advances in pharmacological treatments, the need for novel therapeutic approaches remains [2]. Sodium–glucose cotransporter 2 inhibitors (SGLT2is) have emerged as a promising class of drugs, initially developed as oral hypoglycaemic agents that subsequently demonstrated beneficial effects on HF beyond glycaemic control [3,4,5,6,7]. Recent clinical trials have shown that SGLT2is improve cardiovascular outcomes and reduce the risk of HF hospitalisations and cardiovascular death in patients with HF and reduced ejection fraction (HFrEF) [8,9]. The favourable effects observed with the administration of these agents in HFrEF patients can be attributed to multifaceted mechanisms of action, some of which remain incompletely elucidated [10,11].

Due to the accumulated evidence, SGLT2is have now emerged as a first-line pharmacological approach in patients with HF, irrespective of their left ventricular ejection fraction (LVEF). Following the release of the 2021 heart failure guidelines and the 2023 update, the utilisation of SGLT2is has been endorsed with a class Ia recommendation level [12,13].

Despite the wealth of evidence derived from randomised clinical trials, the current body of real-world data pertaining to the utilisation of this drug class and its safety profile remains limited.

The aim of our study was to evaluate the safety and tolerability profile of dapagliflozin along with other HF drugs in the first year of drug utilisation and to assess the markers of clinical improvement in a real-world population of HFrEF patients. 

## 2. Materials and Methods

### 2.1. Study Design and Data

This retrospective multicentre study involved nine cardiology departments in the Marche region. Written informed consent was obtained from all patients included in the study. In accordance with local legislation and institutional requirements, ethical review and approval were not required for this study involving human participants due to its retrospective nature and the use of de-identified patient data. 

We included outpatients with chronic HFrEF (LVEF ≤ 40%) who had initiated dapagliflozin in an ambulatory setting since January 2022 and had taken the medication for at least 6 months by December 2022. Patients started on dapaglifozin during acute HF hospitalisation and those with type 1 diabetes, an estimated glomerular filtration rate (eGFR) of ≤ 25 mL/min/1.73 m^2^, and with an LVEF of > 40% were excluded. 

For each patient, comprehensive medical histories, encompassing the aetiology of the cardiac disease, risk factors and medications, echocardiographic data, and key laboratory tests at the time of dapaglifozin initiation, were retrieved. After 1 and/or 3 months, a telephone interview was conducted with each patient to assess the occurrence of adverse effects and re-evaluate their renal function and potassium levels. During the six-month follow-up visit, echocardiographic parameters, laboratory findings, and the occurrence of adverse events were re-analysed.

### 2.2. Tolerability Profile 

We evaluated the clinical amelioration during treatment by recording

− Improvement in overall self-reported subjective well-being, considering factors such as symptom relief, quality of life, and general satisfaction with health status.
− Improvement in functional capacity, measured through exercise tolerance, activity levels, and performance in daily tasks.
− Improvement in dyspnoea (according to NYHA classification)
− Weight reduction
− Decrease in natriuretic peptide (NP) values of ≥ 50% from baseline levels
− Ability to up titrate betablockers (BB) and/or angiotensin receptor–neprilysin inhibitors (ARNI) and/or decrease furosemide doses

A score for objective improvement was calculated as a composite of the following measures: weight loss, NYHA decrease, NP decrease ≥50%, and improved GDMT titration. Patients were considered to be improved if they met at least two of the four criteria defined above at the end of the individual follow-up.

### 2.3. Statistical Analysis 

Categorical variables are presented as frequencies and percentages, and differences were tested by the χ^2^ test. Continuous variables are presented as mean and SD or median (interquartile range) and compared by Student’s *t* test or Kruskal–Wallis, according to normality checked by Shapiro–Wilks test. 

Cox multivariable logistic regression was used to test the association of demographics (age and sex) and baseline clinical characteristics (duration of HF, diabetes or prediabetes, and eGFR and Hb levels) with the composite improvement score. Each component is presented as a hazard ratio (HR) and 95% confidence intervals (CI), using *p* < 0.1 in the univariate analyses for inclusion. The significance threshold was set at *p* = 0.05 (2-tailed). All analyses were performed using SPSS for Windows (version 20; SPSS, Inc., Chicago, IL, USA).

## 3. Results

### 3.1. Patient Characteristics

Table 1 presents the demographic and clinical characteristics of the study population. A total of 95 patients were included in the analysis, with a mean age of 66 ± 12 years, and the majority (82%) were men. HFrEF was secondary to ischemic heart disease in 48% of patients. Only 20% of patients had overt diabetes and 14% had an impaired glucose tolerance. Overweight was observed in 43% of the population, with 16% classified as stage 1 (BMI 30–34.9 kg/m^2^) and 10% as stage 2 obesity (BMI 35–40 kg/m^2^). Atrial fibrillation was documented in 22% of the patients. Overall, 47% of the patients had an implantable cardioverter-defibrillator (ICD) and 12% had received cardiac resynchronisation therapy with defibrillator (CRT-D). Furthermore, 22% of the patients were classified as NYHA class III–IV, indicating severe symptoms of heart failure.

### 3.2. Safety and Tolerability Profile 

We recorded 36 adverse clinical events over 6 months in 26 patients (Table 2), with the majority comprising hypotension, and 10 instances of worsening renal function (eGFR decline > 30%). Overall, 5 patients developed three or more adverse events, 6 incurred two untoward effects, 15 developed a single event, and 69 none.

**Figure 1:**
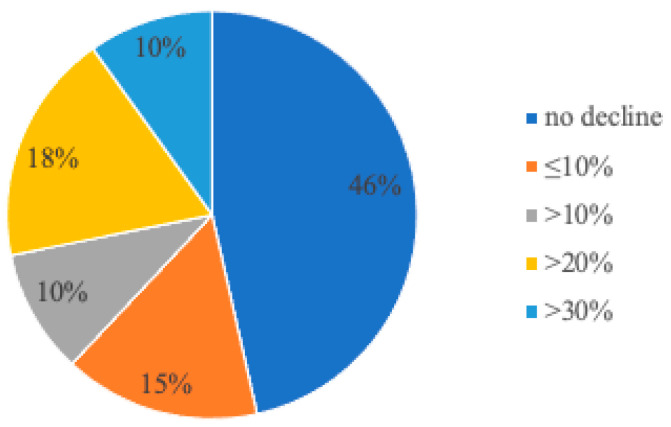
Distribution of adverse changes in renal function defined as percentage decrease in estimated glomerular filtration rate (eGFR) from baseline to 6 months. Although renal function was stable in the majority of patients, 10% developed a significant (≥30%) decline in eGFR.

The distribution of worsening renal function by 6 months is shown in Figure 1. Only two patients manifested a decrease of >50%.

**Figure 2:**
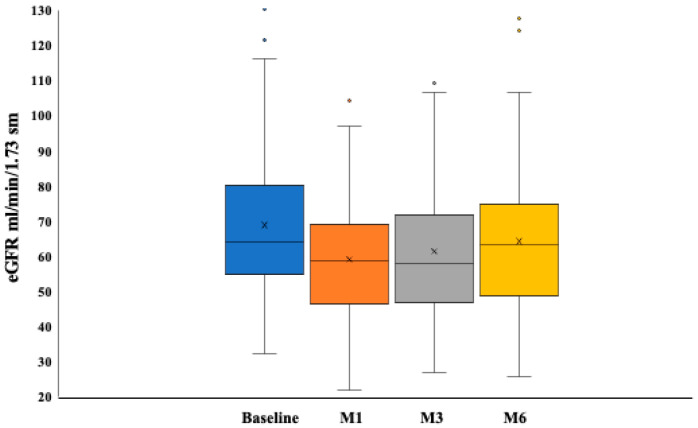
Changes in estimated glomerular filtration rate over the study period. Data are expressed as median and interquartile range of eGFR values. The decline in renal function peaked in the first month after dapaglifozin start and eventually recovered.

The analysis of renal function over time documented nadir eGFR values at the first month test, with an average dip of −5 ± 11 mL/min/1.73 m^2^. eGFR recovered eventually over time, although not completely, by 6 months (Figure 2).

Table 3 provides a comprehensive overview of the key changes observed at 6 months from baseline, encompassing the biochemical, echocardiographic, clinical parameters, and drugs.

**Figure 3:**
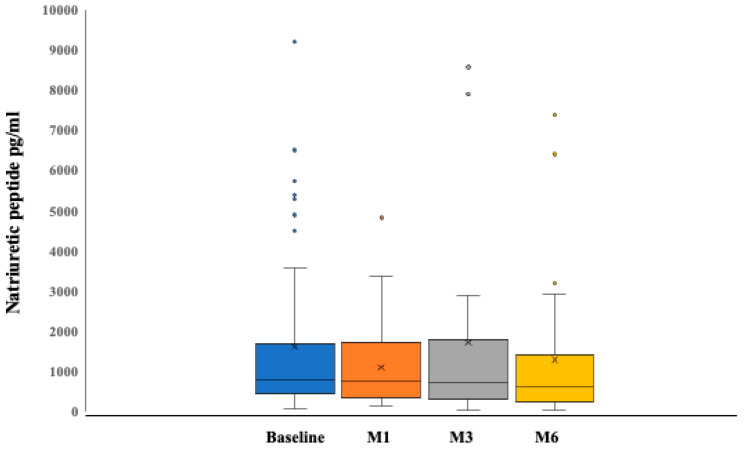
Changes in natriuretic peptides over the study period. Data are expressed as median and interquartile range of eGFR values. A slow decline in NP was observed from month 3 onwards in the first month after dapaglifozin start and eventually recovered.

**Figure 4:**
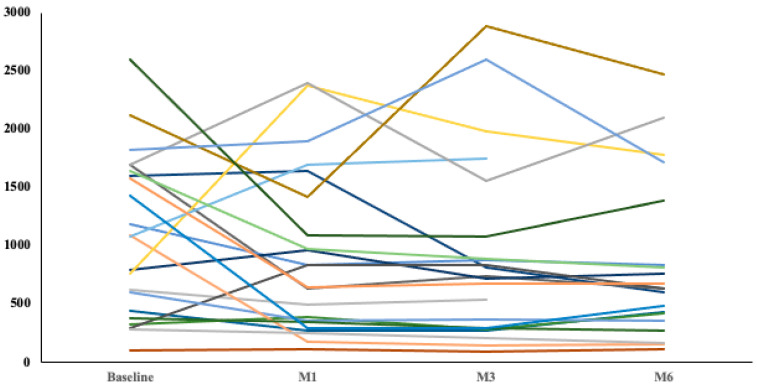
Natriuretic peptide changes over the study period. Individual trends are plotted for all patients with data available at each time interval. Most patients experienced a sustained decline in NP levels.

NP levels decreased, on average, by 23% by 6 months (*p* < 0.001) (Figure 3), and individual trends documented a sustained decline over time in 75% of the patients with multiple data points available (Figure 4).

Echocardiographic measurements revealed a significant decrease in pulmonary artery pressure (PAPs) (*p* < 0.001) and E/e’ (*p* < 0.001), while tricuspid annular plane systolic excursion (TAPSE) was unchanged (*p* = 0.72).

In terms of drug therapy, furosemide dosage decreased significantly (*p* = 0.001), and the percentage of the target dose achieved for angiotensin receptor–neprilysin inhibitors (ARNI) increased significantly (*p* = 0.003). On the other hand, no significant changes were observed in the percentage of the target achieved for beta-blockers (BB) (*p* = 0.42) and mineralocorticoid receptor antagonists (MRA) (*p* = 0.84).

**Figure 5:**
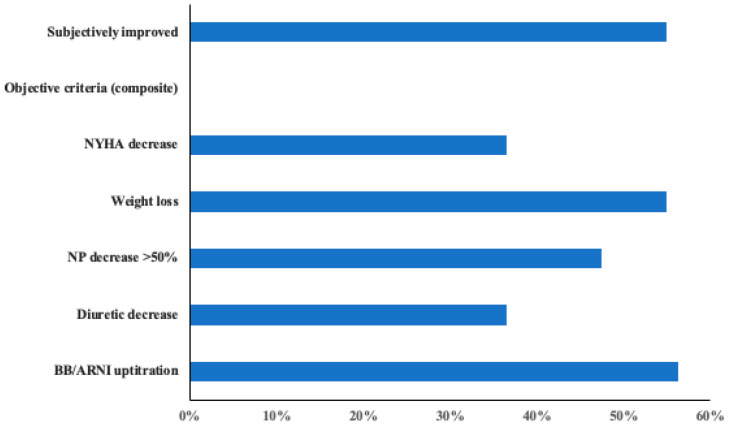
Proportion of patients experiencing beneficial effects.

Figure 5 depicts the overall incidence of subjective improvement and objective favourable changes observed during follow-up.

Overall, 39 patients improved based on the composite score of objective measures. By multivariable Cox regression, after adjustment for age, sex, the presence of diabetes or prediabetes, the duration of HF, higher Hb concentrations (HR 1.347, 95% CI 1.038–1.746, *p* = 0.025), and higher eGFR levels (HR 1.016, 95% CI 1.000–1.033, *p* = 0.046), these variables at baseline remained independently associated with the composite improvement score.

## 4. Discussion

In this real-life experience of dapagliflozin utilisation in HFrEF patients, the drug demonstrated a high level of safety and tolerability, with few serious adverse events, and was associated with global clinical improvement, an NP decrease, and a better GDMT uptake over 6 months.

The clinical characteristics of our study cohort, including the preponderance of male patients, as well as the prevalence of ischemic aetiology (almost 50% of patients) and high rate of device implants, are consistent with the distribution commonly observed in HFrEF populations in cardiology settings [14].

Moreover, 22% of our patients had atrial fibrillation, which adds complexity to the management of HFrEF and underscores the need for interventions that can address both conditions effectively [15].

Although the proportion of patients with diabetes was low (20%) and consistent with findings from previous randomised clinical trials [8,9], the safety profile in terms of hypoglycaemia was sustained and the utilisation of dapagliflozin did not increase the risk of hypoglycaemic episodes in this subset of patients [16].

One of the most intriguing findings of our study is that the addition of dapagliflozin to the treatment regimen facilitated a smoother titration of sacubitril/valsartan. Sacubitril/valsartan is a guideline-directed therapy that has been shown to improve outcomes in HF patients [17,18]. However, achieving optimal dosing can be challenging due to the risk of hypotension and the need for close monitoring during titration [19]. At present, there is no existing research providing conclusive evidence that the introduction of SGLT2is facilitates the titration of ARNI. Furthermore, there is limited evidence regarding the concurrent use of sacubitril/valsartan and dapagliflozin, as the major randomised trials investigating the use of dapagliflozin included a relatively small proportion of patients on ARNI (approximately 10%) [8]. Both medications have demonstrated beneficial effects on cardiovascular outcomes and have complementary mechanisms of action [20]. The introduction of dapagliflozin to our treatment approach appeared to simplify the sacubitril/valsartan titration, allowing for more efficient and effective optimisation of this medication.

In our study, we observed a significant reduction in furosemide doses, which can be attributed to the decongestive effects of dapagliflozin and may also be partly explained by the concomitant titration of sacubitril/valsartan. The guidelines for HF management emphasise the prioritisation of titrating the “fabulous four” (SGLT2is, ARNI, beta-blockers, and MRAs), while the addition of diuretics should be considered only if the pharmacological regimen is insufficient to maintain a state of decongestion. [12,13]. The diuretic effect of dapagliflozin is well-documented, as it promotes natriuresis and reduces fluid overload in heart failure patients [21]. By enhancing renal sodium and glucose excretion, dapagliflozin contributes to a more favourable volume status, thereby potentially reducing the reliance on loop diuretics [22]. Furthermore, the successful titration of ARNI may have contributed to the reduction in furosemide doses observed in our study. Sacubitril/valsartan improves neurohormonal modulation and cardiac remodelling, leading to improved heart failure outcomes [23]. By addressing the underlying pathophysiological mechanisms of HF, sacubitril/valsartan may help to achieve decongestion and reduce the need for diuretic therapy [24,25].

The analysis of baseline and 6 month follow-up data revealed a significant decrease in BMI, reflecting the potential impact of SGLTi therapy on weight reduction [26], which has been primarily associated with a decrease in fat mass [27] without affecting muscle mass [28].

Consistent with previous randomised trials [29], the addition of dapagliflozin in our cohort did not result in any significant changes in blood pressure values at the six-month follow-up.

Regarding the echocardiographic and laboratory parameters, our data analysis demonstrated a significant reduction in E/e’ ratio and pulmonary artery pressure (PAPs) at six months, aligned with a significant reduction in natriuretic peptide levels. Currently, the literature provides evidence from studies conducted on small patient cohorts, suggesting that the addition of SGLT2is can improve LVEF through favourable left ventricular remodelling, while randomised studies have shown only a mild reduction in natriuretic peptide levels associated with the use of SGLT2 inhibitors [30,31,32]. In a recent study [33] on 162 patients with HF, the addition of dapagliflozin was associated with a reduction in both atrial and ventricular volume, with no change in doppler filling pressures, but a significant NT-proBNP concentration reduction at 30 days. Our findings of reduced filling pressures and PAPs and decreased NP levels may be attributed to both the diuretic effect of dapagliflozin and the concurrent titration of sacubitril/valsartan. Indeed, findings from the PARADIGM-HF trial have firmly established that sacubitril/valsartan has the capacity to reduce NP levels [24]. An NP decrease may be indicative of enhanced cardiac function and reduced volume overload, which are goals of both sacubitril/valsartan and dapagliflozin therapy [34].

Dapagliflozin has been shown to exert favourable effects on myocardial metabolism and function. Preclinical studies suggest that SGLT2 inhibition may enhance myocardial energetics by promoting the utilisation of ketone bodies as an alternative fuel source for the heart [35]. Ketone bodies are efficient substrates for ATP production, particularly in conditions of metabolic stress such as heart failure. By shifting the cardiac metabolism away from reliance on glucose and fatty acids, dapagliflozin may improve myocardial efficiency and function. Additionally, dapagliflozin exhibits cardioprotective effects through its modulation of neurohormonal pathways and inflammatory processes. Studies have reported reductions in markers of oxidative stress, inflammation, and fibrosis following treatment with SGLT2 inhibitors [36]. These pleiotropic effects may contribute to the improvements in cardiac remodelling, endothelial function, and myocardial fibrosis [37]. Given the limited sample size in our study, no speculative hypotheses regarding a left ventricular remodelling effect can be proposed.

With respect to renal function, our data analysis showed a significant increase in creatinine values at six months, coupled with a concurrent, albeit modest, decrease in glomerular filtration rates. This early decline in renal function has been proposed to stem from haemodynamic alterations, such as decreased intraglomerular pressure and efferent arteriolar vasoconstriction, which could lead to a temporary reduction in GFR. Nevertheless, it is essential to emphasise that these changes are transient and, eventually, renal function becomes more stable over time during ongoing dapagliflozin therapy [38]. Despite the different patient setting, these findings are consistent with the results of the DAPA-CKD study [39], where the use of dapagliflozin was associated with an initial decline in renal function indices during the first 4–8 months of treatment, followed by an eventual improvement, with a significant gain in GFR compared to placebo at 36 months.

The number of adverse events in our study population was low (36 events at 6 months), once again emphasising the safety profile of dapagliflozin, as previously confirmed by randomised studies [40,41]. The most common adverse event was symptomatic hypotension (7 events out of 36), which, however, never led to the discontinuation of dapagliflozin therapy. The management of this adverse event was based on reducing the diuretic dosage, and in cases of persistent symptoms, temporarily reducing the sacubitril/valsartan dosage. Similarly, transient reductions in glomerular filtration rate were managed by reducing the diuretic dose while closely monitoring the patient’s clinical stability and creatinine levels., The composite endpoint of objective improvement at six months was associated with higher baseline Hb concentrations and eGFR levels after adjustment for age, sex, the presence of diabetes or prediabetes and the duration of HF. This suggests that patients with higher haemoglobin and eGFR at the beginning of the study were more likely to respond positively to dapagliflozin therapy. These findings could be valuable for identifying patients who may derive greater benefits from the addition of dapagliflozin to their treatment for HFrEF. Our real-world findings should be interpreted considering the perspective of previous evidence, including DAPA-HF on 4747 patients with HFrEF, where baseline kidney function, dichotomized according to an eGFR greater or less than 60 mL/min/m^2^, did not alter the advantages of dapagliflozin concerning morbidity and mortality in HFrEF [38]. Nevertheless, it is important to note that dapagliflozin’s effectiveness depends on various factors, including individual clinical profiles and the presence of comorbidities. Further clinical studies are required to validate these findings and gain a better understanding of the interplay among predictive factors for dapagliflozin response.

Our study has several limitations: the limited sample size, the relatively short follow-up period of 6 months, and the predominance of male patients, which could reduce the generalisability of the findings to the broader population. Additionally, the study design relied on observational data, potentially introducing biases and limitations inherent to non-randomised studies. While our study provides valuable insights into the real-world utilisation and safety profile of dapagliflozin in patients with HFrEF, several avenues for future research warrant exploration. Firstly, conducting subgroup analyses to identify the specific real-world patient populations that derive the greatest benefit from dapagliflozin would be valuable. This could involve investigating outcomes based on factors such as age, sex, the presence of comorbidities (e.g., diabetes), baseline renal function, and the severity of heart failure symptoms.

Additionally, further research into the underlying mechanisms of action of dapagliflozin beyond glycaemic control is essential. Understanding how dapagliflozin affects cardiac function, neurohormonal regulation, and vascular physiology could elucidate its multifaceted benefits in heart failure management.

Our data do not allow for a formal economic evaluation that would provide valuable information for healthcare decision makers on the budget implications of incorporating dapagliflozin into routine clinical practice. Previous assessments [42] have documented the cost effectiveness of dapaglifozin for HFrEF among others in countries similar to ours, with comparable costs of treatment and hospital admissions, suggesting that comparable assumptions might be valid for Italy. Exploring these aspects for future research will contribute to a more comprehensive understanding of the role of dapagliflozin in the management of heart failure and facilitate the translation of research findings into clinical practice.

In conclusion, our study demonstrates that dapagliflozin therapy in a real-life population of HFrEF patients is not only safe and well-tolerated, but can also facilitate the titration of prognostic drugs such as sacubitril–valsartan, improving echocardiographic and biomarker parameters of congestion.

## Figures and Tables

**Table 1:** Clinical characteristics of the study population.

Sex, female	17 (18)
Hypertension	54 (57)
Ischemic aetiology	46 (48)
Impaired glucose tolerance	13 (14)
Diabetes	19 (20)
Overweight (BMI ≥ 25 kg/m^2^)	40 (43)
Stage 1 obesity (BMI 30–34.99 kg/m^2^)	15 (16)
Stage 2 obesity (BMI 35–40 kg/m^2^)	9 (10)
Atrial fibrillation	21 (22)
ICD	45 (47)
CRT-D	11 (12)
NYHA class III–IV	21 (22)

Data are expressed as n° (frequency percentage). BMI: Body Mass Index, CRT-D: cardiac resynchronisation therapy-defibrillator, and ICD: implantable cardioverter-defibrillator, NYHA: New York Heart Association.

**Table 2:** Clinical events during follow-up.

	Overall
Atrial fibrillation	1
HF Hospitalisation	1
HF death	1
Fatigue	9
Hypotension	6
Symptomatic hypotension	7
Dyspnoea	5
Angina	1
Bloating	1
Palpitations	1
Urinary Tract Infection	3

HF: heart failure.

**Table 3:** Changes in clinical, laboratory, and drug therapy values after 6 month dapaglifozin.

Variable	Baseline	6 Months	*p*
Body Mass Index kg/m^2^ *	27.3 ± 4.6	27.0 ± 4.4	0.017
Systolic BP mmHg	114 ± 16	112 ± 13	0.27
Haemoglobin g/L	14.3 ± 1.5	14.4 ± 1.6	0.44
Creatinine mg/dL *	1.17 ± 0.3	1.24 ± 0.4	0.028
eGFR mL/min/1.73 m^2^ *	69 ± 21	64 ± 20	0.03
Sodium mEq/L	140 ± 4	140 ± 3	0.51
Potassium mEq/L	4.5 ± 0.5	4.5 ± 0.5	0.39
Natriuretic peptides * pg/mL	778 [425–1688]	648 [268–1320]	<0.001
Echo			
PAPs mm *	32 ± 10	28 ± 7	<0.001
E/e’ *	14 ± 5	10 ± 3	<0.001
TAPSE	20 ± 3	20 ± 3	0.72
Drug therapy			
Furosemide mg/day *	39 ± 34	31 ± 37	0.001
BB (% of target)	51 ± 31	51 ± 32	0.42
ARNI * (% of target)	54 ± 39	63 ± 37	0.003
MRAs (% of target)	41 ± 38	42 ± 39	0.84

Data are expressed as mean ± SD (standard deviation) or median [interquartile range]. * *p* < 0.05; ARNI: angiotensin receptor–neprilysin inhibitors, BB: beta blockers, BP: blood pressure, eGFR: estimated glomerular filtration rate (calculated by CKD-epi formula), MRAs: aldosterone receptor antagonists, PAPs: pulmonary artery systolic pressure, and TAPSE: tricuspid annular plane excursion.

## Data Availability

The original contributions presented in the study are included in the article, further inquiries can be directed to the corresponding author.
